# Present-Day State of Percutaneous Endovascular Aortic Aneurysm Repair

**DOI:** 10.3390/jcm15166381

**Published:** 2026-08-18

**Authors:** Paweł Grzyb, Bartosz Rodziewicz, Joanna Halman, Łukasz Znaniecki, Jacek Wojciechowski

**Affiliations:** 1 Faculty of Medicine, Medical University of Gdańsk, 80-210 Gdańsk, Poland; b.rodziewicz@gumed.edu.pl; 2 Scientific Circle of Neurotraumatology, Department of Emergency Medicine, Medical University of Gdańsk, 80-210 Gdańsk, Poland; 3 Department of Emergency Medicine, Medical University of Gdańsk, University Clinical Centre in Gdańsk, 80-952 Gdańsk, Poland; 4 Vascular Surgery Department, Medical University of Gdańsk, University Clinical Centre in Gdańsk, 80-952 Gdańsk, Poland; lukasz.znaniecki@gumed.edu.pl (Ł.Z.); jacek.wojciechowski@gumed.edu.pl (J.W.)

**Keywords:** percutaneous endovascular aneurysm repair, preclose technique, abdominal aortic aneurysm, femoral artery access, quality of life, cost-effectiveness

## Abstract

Abdominal aortic aneurysm (AAA) carries a rupture-related mortality exceeding 80% when untreated. Percutaneous endovascular aortic aneurysm repair (pEVAR) represents the current technical frontier, although evidence regarding its clinical and economic benefits remains heterogeneous. This narrative review of PubMed, Scopus, and Web of Science from inception to 1 August 2026 synthesises comparative data from 67 publications and four manufacturer websites to evaluate the clinical, quality-of-life, and economic outcomes of pEVAR against open femoral exposure. Current evidence suggests that pEVAR may serve as a feasible alternative to open femoral exposure, offering potential benefits regarding access-site morbidity, hospital length of stay, and early postoperative pain. While RCTs report pEVAR’s early perioperative advantages, evidence regarding long-term safety, anatomy, and costs remains predominantly observational. Additionally, pEVAR allows for locoregional anaesthesia, a strategy hypothesized to positively influence short-term outcomes in ruptured aneurysm emergencies, although prospective validation is required. However, maintaining these benefits relies heavily on overcoming the procedural learning curve and ensuring rigorous pre-procedural patient selection via computed tomography angiography to avoid severe vessel calcification and excessive depth. Available evidence suggests that in carefully selected patients, pEVAR yields an overall safety profile comparable to surgical cutdown, while potentially mitigating the risk of access-site complications.

## 1. Introduction

An abdominal aortic aneurysm is a degenerative condition characterised by the dilation of the infrarenal aorta to at least 1.5 times its normal size. Although its incidence has decreased secondary to improved cardiovascular management and lower smoking rates, population screening still shows a prevalence of 1.4–7% among individuals between 50 and 84 years old [1,2]. Given that a ruptured AAA carries a mortality rate exceeding 80%, elective repair is recommended once the sac diameter reaches 55 mm in men or 50 mm in women [1,2,3,4]. Historically, open surgical repair served as the standard of care, though it was associated with a significant perioperative mortality rate even in high-volume centres.

The management of AAA evolved significantly in the late 20th century, with the development of endovascular techniques. Initially performed by Volodos in 1987 and introduced broadly by Parodi in 1991, endovascular aortic repair (EVAR) was developed as an alternative for patients considered high-risk for open repair [5,6]. Accessing the aorta transluminally via the femoral arteries established a less invasive pathway for these individuals [7,8]. Randomised controlled trials, including EVAR-1, DREAM, OVER, and ACE, demonstrated early perioperative mortality advantages for EVAR, although this survival advantage tends to level out over five to ten years due to the need for ongoing device surveillance [7,9,10,11]. Following iterative improvements in stent-graft technology and improved safety profiles, EVAR has largely replaced open surgery as the primary intervention for anatomically suitable patients.

Despite intra-aortic repair being minimally invasive, early EVAR required cutdown surgical access (cEVAR). First-generation stent-grafts required large-bore delivery systems, up to 24 French in size, necessitating surgical exposure of the common femoral artery (CFA). However, open groin cutdowns are inherently associated with access-site complications, including surgical site infections, lymphoceles, haematomas, nerve injury, and delayed ambulation, which contribute to total procedural morbidity and extended hospital stays [12,13].

The biological substrate of aortic disease, characterised by extracellular matrix remodelling and elastic fibre disruption, drives continuous aneurysm progression [14]. This structural heterogeneity also dictates postoperative evolution independently of procedural success. Because the severity of these histopathological changes predicts overall mortality, it necessitates individualised postoperative management and surveillance [15]. Consequently, evaluating the efficacy of interventions such as pEVAR requires integrating baseline biological characteristics with technical outcomes.

A fully percutaneous approach (pEVAR) was introduced in 2002, enabled by the miniaturisation of delivery systems (14–20 French) and the introduction of suture-mediated closure devices (such as Perclose ProGlide) applied via the “preclose” technique [16,17,18]. This method allows arterial closure via percutaneous puncture, avoiding open groin exposure in anatomically eligible candidates. Early data showed a 92% closure success rate and a 53% drop in access complication risks compared to open cutdowns [19]. In contemporary vascular practice, pEVAR achieves successful haemostasis in up to 98% of cases, yielding a decrease in local access-site complications [16]. Subsequent studies suggest that pEVAR yields a clinical safety profile comparable to cEVAR, while potentially reducing local access-site morbidity, shortening procedural time, and facilitating recovery through reduced tissue trauma and the use of local or regional anaesthesia pathways [20,21,22,23,24].

Although previous studies highlight the potential advantages of pEVAR in terms of postoperative recovery, length of hospital stays, and early patient comfort, patient-centred and economic outcomes remain inconsistently reported across the literature. Most available studies primarily focus on technical success and access-related complications, while quality-of-life measures and formal cost-effectiveness analyses are often secondary endpoints or are assessed using heterogeneous methodologies. Recently published evidence, including the SWEET-EVAR randomised controlled trial, provided additional evidence regarding patient-centred outcomes following pEVAR, including recovery time, pain, and early quality-of-life measures. Therefore, we considered an updated review incorporating these contemporary data to be clinically justified. Despite growing evidence supporting the minimally invasive nature of pEVAR, the broader clinical and economic impact of this approach compared with surgical femoral cutdown remains insufficiently characterised.

## 2. Materials and Methods

Our process was structured around the five foundational components of narrative knowledge synthesis proposed by Sukhera: providing a clear methodological rationale; setting explicit conceptual boundaries; justifying literature selection criteria; practising author reflexivity to determine thematic sufficiency; and clearly detailing our interpretive analysis [25].

### 2.1. Search Sources and Strategy

A literature search was conducted across PubMed, Scopus, and Web of Science from inception to 1 August 2026. Boolean operators were used, and in the case of PubMed, MeSH terms were included.

To maximize sensitivity and ensure a broad retrieval of comparative data, the search string combined core procedural terms, access modalities, and specific device names.

The primary search string combined the terms percutaneous endovascular aneurysm repair”, “pEVAR”, “percutaneous access”, “totally percutaneous”, “femoral access”, “percutaneous EVAR”, “femoral cutdown”, “open femoral exposure”, “preclose”, “aneurysm”, “percutaneous endovascular aortic repair”, “vascular closure device”, “ProGlide”, “Prostar”, and “MANTA”, supplemented with domain-specific keywords such as “cost”, “quality of life”, “anaesthesia”, “calcification”, and “predictor”. Full search strings for each database are provided in Supplementary Material File S1. Furthermore, a grey literature search was conducted.

### 2.2. Eligibility Criteria

The specific parameters guiding the literature search and study inclusion are detailed below.

Inclusion Criteria: Randomised controlled trials (RCTs), prospective non-randomised comparative studies, retrospective cohort studies, and cost-minimisation analyses from comparative cohorts with original or contextual data.

Exclusion Criteria: Case reports, case series, conference abstracts, letters to the editor, and editorials without relevant contextual data. Systematic reviews, meta-analyses, and manufacturer data were used for contextual background only.

### 2.3. Study Selection

Title and abstract screening, full-text eligibility assessment, and data extraction were performed by two reviewers (P.G. and B.R.). Any disagreements between the primary reviewers were resolved by a third reviewer (J.H.).

**Figure 1:**
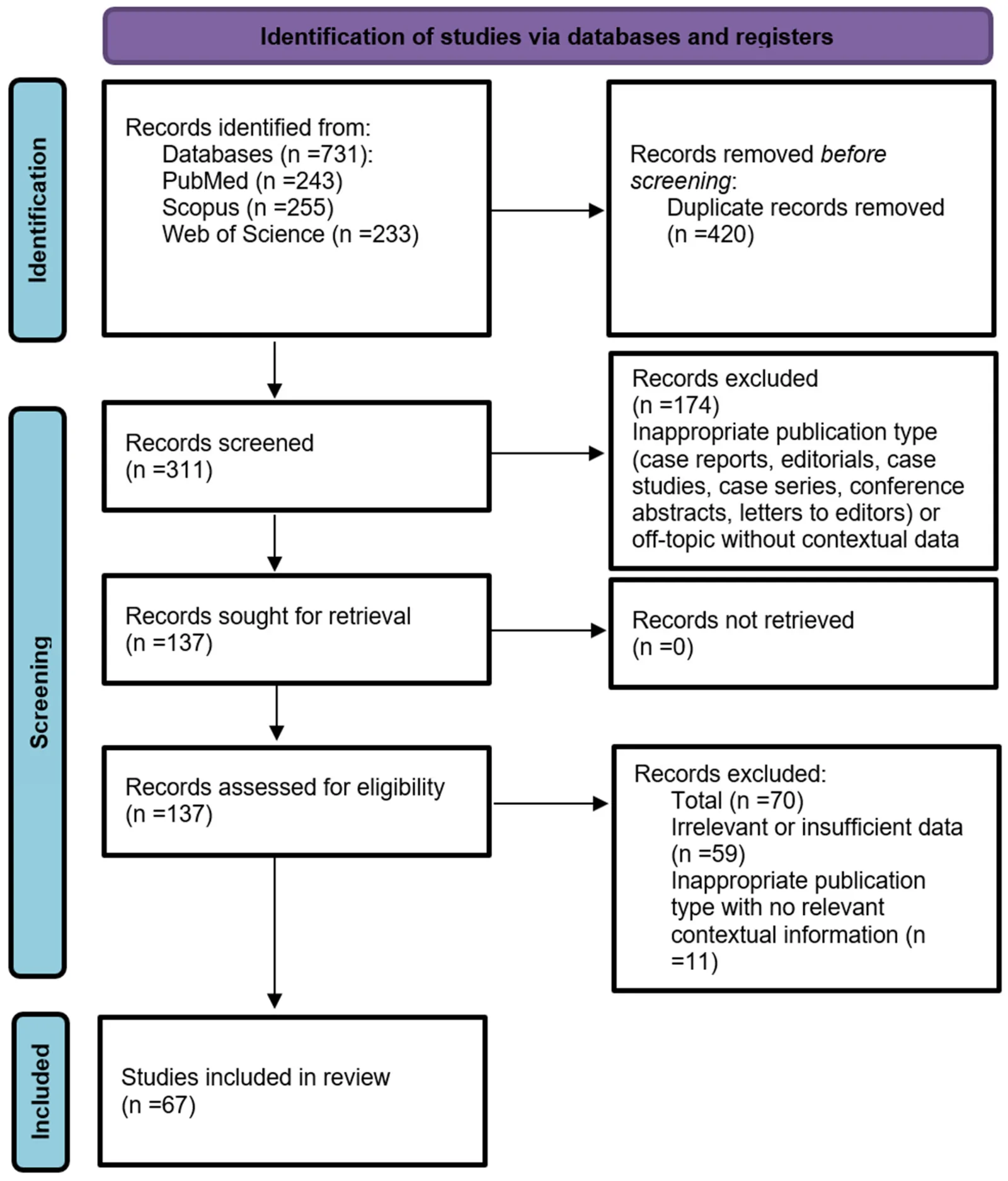
This flowchart is illustrative and shows the study selection process [26].

The initial database search retrieved 731 records. After the removal of 420 duplicates, 311 titles and abstracts were screened, resulting in the exclusion of 174 records that did not meet the fundamental inclusion criteria. The remaining 137 full-text articles were assessed for eligibility, focusing on clinical outcomes, quality of life, and economic endpoints. Of these, 70 were excluded primarily due to a lack of comparative access modality, missing relevant clinical or economic endpoints, or inappropriate study design (systematic reviews and meta-analyses were excluded from the primary data synthesis to avoid duplicate patient counting, though they were retained for contextual background). Following full-text screening, 67 publications met the relevant criteria and were included in the final review. The complete selection process is detailed in Figure 1. Additionally, four manufacturer websites were accessed for device specifications. A summary identifying all studies and records included in the review is depicted in Supplementary Material Table S1.

## 3. Results

### 3.1. Procedural Mechanics and Technique

**Figure 2:**
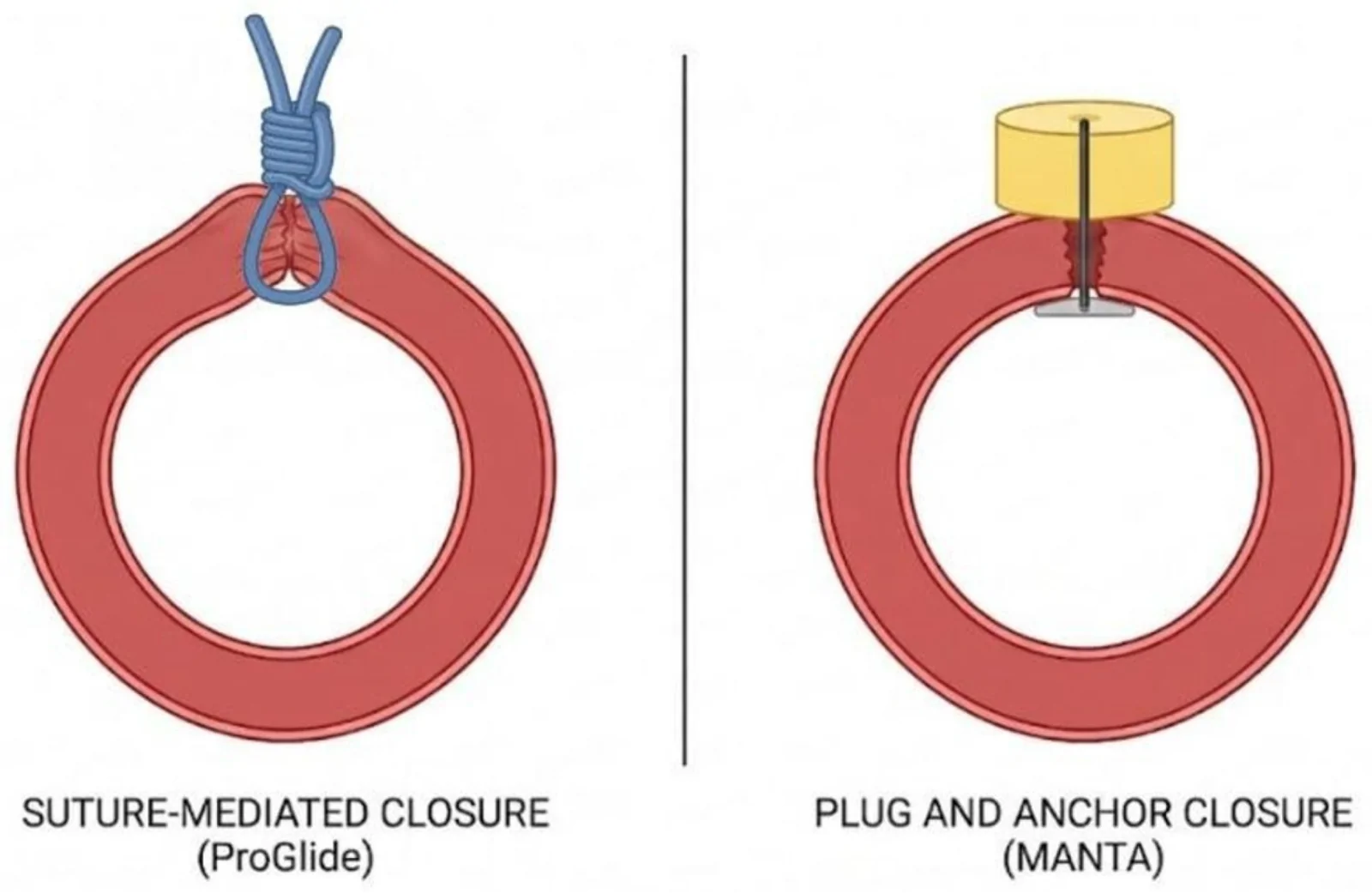
Comparison between suture-mediated closure and plug and anchor closure. Created in BioRender. Rodziewicz, B. (2026) https://biorender.com/nwq11f3 (accessed on 3 July 2026).

The foundation of standard pEVAR is the “preclose” principle. Because primary suture deployment following large-bore arterial sheath removal (up to 20 French) is technically complex, suture-mediated closure devices (such as the ProGlide) are deployed into the common femoral artery (CFA) before the large working sheaths are introduced. Meta-analytic evidence further corroborates that while the preclose technique achieves a 94% technical success rate per arterial access, technical failure increases with larger sheath sizes (≥20F) and limited operator experience, whereas routine ultrasound guidance enhances procedural success [27]. Beyond large-bore access, the efficacy of ultrasound-guided vascular closure in common femoral artery punctures is well documented in broader peripheral endovascular interventions. In a retrospective cohort of 301 patients undergoing antegrade access with 6–8 Fr sheaths, ultrasound-guided deployment of the FemoSeal device achieved a 99% technical success rate with a low rate of pseudoaneurysm formation (1%), further corroborating the utility of real-time imaging guidance in minimizing local access site morbidity during percutaneous femoral closure [28]. While the dual-ProGlide method is standard, alternative closure strategies and devices have been increasingly adopted. For example, the MANTA device, which deploys a collagen plug post-procedure (a comparison of suture-mediated and plug and anchor closure is presented in Figure 2), demonstrates a success rate of approximately 98% with a single insertion [29].

Recent evidence indicates that both suture-mediated and plug-based vascular closure devices deliver effective and comparable outcomes for large-bore access during endovascular aneurysm repair. Final successful haemostasis is achieved at nearly identical rates for both devices—91.8% for ProGlide and 90.5% for MANTA [30]. Consequently, the rates of failure necessitating open surgical conversion are statistically similar between the two cohorts [30].

However, the operative pathways to achieve closure often differ. The use of the ProGlide device is characterised by a significantly higher reliance on adjunctive measures, such as prolonged manual compression, covered stent usage, or deployment of a secondary closure device, to secure complete haemostasis compared to the MANTA plug [30]. Regarding postoperative safety, neither device is associated with significant major occlusive complications. Pseudoaneurysm formation represents the primary minor complication, occurring at statistically similar frequencies between the groups, although the risk is elevated in cases where operators rely on secondary adjunctive measures to salvage the initial closure [30]. Numerical data comparing the specific success rates, complication frequencies, and procedural requirements of the ProGlide and MANTA systems are compiled in Table 1. Furthermore, systematic reviews evaluating Food and Drug Administration (FDA)-approved VCDs suggest that overall safety and complication rates remain comparable to manual compression (12% vs. 13%), highlighting that device efficacy varies according to patient anatomy and clinical scenarios, which warrants a tailored patient-selection strategy [31].

In selected cases or in centres without access to specialised closure devices, the fascial suture technique remains a viable fallback [32,33]. Even complex thoracic or branched repairs requiring sheaths larger than 21 French can achieve 94–95% percutaneous success rates in experienced hands, provided strict anatomical criteria are met [34,35]. A comparison of commonly used vascular closure devices (VCDs) and approximate device costs is shown in Table 2.

Regarding suture-mediated devices, meta-analytic data report that ProGlide is associated with a lower risk of overall bleeding (RR 1.82) and bleeding complications (RR 2.48) compared to older-generation systems such as Prostar XL [42].

### 3.2. Head-to-Head Comparison with Open Femoral Exposure

The possible clinical advantages of pEVAR over traditional surgical cutdown have been evaluated through granular procedural metrics in randomised trials. In the PEVAR trial, the percutaneous approach achieved a 94% technical success rate while demonstrating measurable intraoperative benefits [20]. Specifically, pEVAR reduced the mean overall procedure time by 34 min and the time to achieve haemostasis by approximately 10 min compared to surgical exposure [20]. Furthermore, the trial reported that patients treated percutaneously required less total anaesthesia time, experienced reduced blood loss, and had a lower need for intraoperative transfusions [20]. These time differences are closely associated with the access site preparation; a prospective crossover study demonstrated that achieving percutaneous access requires less time than a surgical cutdown (mean of 11.5 min versus 24.8 min) [43].

Regarding postoperative recovery, the avoidance of a groin incision fundamentally influences early recovery metrics. In the aforementioned crossover study, where patients received pEVAR in one groin and surgical cutdown in the contralateral groin, the percutaneously accessed side was scored as significantly less painful on both the first and fifth days following surgery [43]. The SWEET-EVAR trial further quantified this metric, reporting that pEVAR patients experienced a shorter total duration of pain (4 days versus 8 days) compared to the cutdown cohort [21]. Consequently, patients required fewer analgesics upon discharge and returned to their normal daily activities sooner (16 versus 28 days), which correlated with higher health-state utility scores at two weeks post-operation [21].

While the previously mentioned RCTs provide high-quality evidence supporting acute procedural advantages and early recovery metrics, the evaluation of rare access-site morbidity and population-level readmission rates is largely dependent on observational data.

For instance, in larger population cohorts, these perioperative advantages translate into reductions in hospital readmissions and access-related morbidity. A retrospective cohort study of 14,002 EVAR patients from the American College of Surgeons NSQIP database indicated that a successful percutaneous approach is associated with an unplanned 30-day readmission rate of just 5.7% [44]. In contrast, undergoing bilateral cutdowns demonstrated a 7.6% readmission rate, primarily driven by access-site morbidity [44]. The analysis reported that bilateral surgical cutdowns carried an adjusted odds ratio of 4.41 for wound complications compared to pEVAR [44]. Furthermore, the data indicate that a single incision, whether a planned unilateral cutdown or a failed percutaneous attempt converted to open surgery, increases the risk of wound complications and readmissions, supporting the clinical rationale for attempting a percutaneous procedure when anatomical criteria are met [44].

Beyond controlled trials and specific databases, multicentre registries report comparable success rates along shorter hospital stays [45,46]. In the setting of ruptured AAA emergencies, observational data suggest that pEVAR achieves closure success rates comparable to elective settings and facilitates the use of locoregional anaesthesia [47,48].

### 3.3. Risk Factors for pEVAR Failure

Success relies partially on pre-procedural CT angiography for appropriate patient selection. Favourable anatomical parameters include a CFA diameter of at least 6 mm, a skin-to-artery depth under 4 cm, a safe puncture zone well above the bifurcation, and less than 50% circumferential wall calcification (especially on the anterior surface) [24,45,49,50,51]. To optimise safety, the initial puncture of the anterior CFA wall is routinely performed under ultrasound guidance [51].

**Figure 3:**
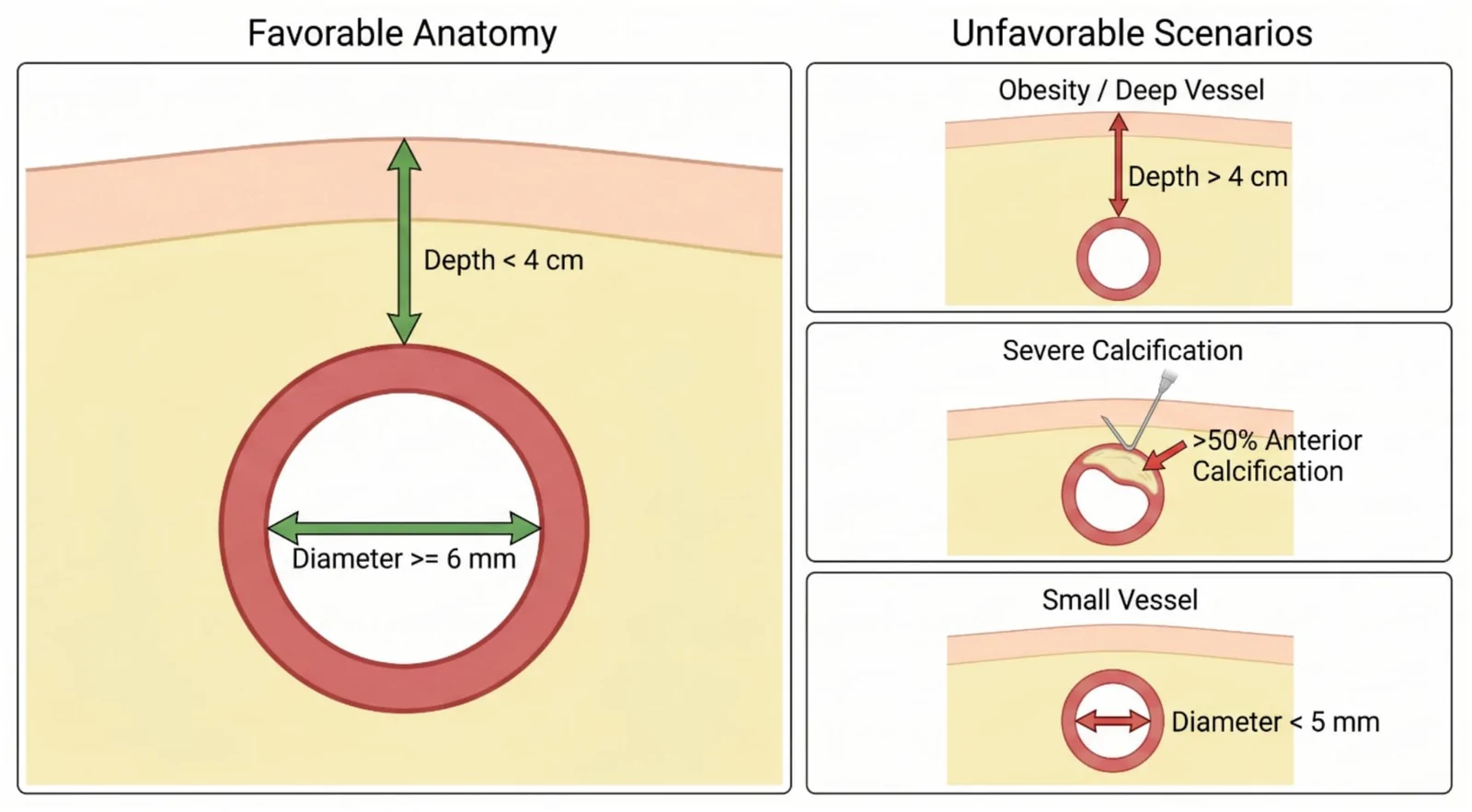
Comparison between favourable and unfavourable anatomical scenarios for pEVAR. Created in BioRender. Rodziewicz, B. (2026) https://BioRender.com/rm3f2as (accessed on 3 July 2026).

In cases of pEVAR failure, specific anatomical variables are identified as primary predictors. The most reliable predictor of technical failure is severe anterior femoral artery calcification. Extensive calcific plaque impedes the closure device’s needles from passing through the tissue and securing a knot [45,49,52]. Another major anatomical barrier is obesity-related vessel depth. When the CFA sits deeper below the skin, it may alter the deployment angle of the closure device, making failure significantly more likely [50]. In such challenging anatomies, including scarred groins, a minimal 2–3 cm surgical cutdown provides direct vascular control, effectively bypassing these technical barriers without significantly increasing perioperative morbidity. Finally, attempting percutaneous closure in small-calibre arteries (under 5 mm) is generally not recommended, and cEVAR access should be considered [51]. These anatomical scenarios are depicted in Figure 3.

Beyond anatomical factors, operator experience is a major determinant of success. Data show that the probability of technical failure is inversely correlated with the surgeon’s skills, where it drops from roughly 45% to 5% per patient [53].

Furthermore, while female patients typically present with smaller, deeper, and more calcified femoral arteries, applying strict preoperative CT selection criteria allows operators to achieve technical success rates of 96% in carefully selected female candidates [54]. However, it should be noted that contemporary systematic evidence incorporating over 500,000 EVAR procedures demonstrates that female sex is independently associated with smaller-calibre access vessels and a significantly increased risk of 30-day mortality (adjusted OR 1.73, 95% CI 1.32–2.26), in-hospital mortality (OR 1.90, 95% CI 1.43–2.53), and early access-related morbidity [55,56]. Women face a higher risk of lower limb ischaemia (OR 2.44, 95% CI 1.73–2.43; 5.3% vs. 3.2% in registries) and higher rates of major periprocedural bleeding (22.0% vs. 15.9%) [55,56].

Consequently, access suitability must be systematically evaluated along the entire iliofemoral axis—taking into account vessel diameter, circumferential calcification, and tortuosity—rather than restricting pre-procedural assessment solely to the common femoral artery puncture site [55,56]. In selected patients with small, heavily calcified, or otherwise hostile access vessels, attempting percutaneous closure may be ill-advised; in such scenarios, planned surgical exposure, an iliofemoral conduit, or a hybrid access strategy may provide more immediate vascular control and facilitate the prompt management of intraoperative complications, including arterial rupture, dissection, acute thrombosis, or closure-device failure [55,56].

### 3.4. Cost-Effectiveness

The financial viability of pEVAR depends on the trade-off between upfront device costs and downstream hospital savings. While closure devices increase direct procedural costs, pEVAR is generally considered cost-saving when compared to cEVAR [22]. The economic balance favours pEVAR because percutaneous access significantly reduces operating theatre time, minimises the need for expensive wound care, and ensures that patients are discharged faster. Decision-tree modelling indicates mean savings of approximately USD 751 per procedure, although this estimate was derived from a single US healthcare network and may not be transferable to other reimbursement systems [22,57]. However, appropriate patient selection remains the main economic determinant—if a percutaneous attempt fails and requires intraoperative conversion to cutdown, the hospital absorbs both the combined costs of the deployed closure devices and the extended surgical time [23]. When executed successfully on properly selected patients, pEVAR facilitates safe, same-day ambulatory discharge, compounding hospital savings without increasing complication or readmission rates [58,59,60,61].

In terms of hospital resource allocation, a recent comparative study highlighted that pEVAR significantly reduces both the overall operative duration (mean of 141.4 min versus 218.5 min for cutdown) and the need for intensive care unit (ICU) admissions (40% versus 66.7%) [62]. These operational time and readmission reductions are vital, especially in patients with low perioperative risk, as pEVAR can sometimes result in negative operating margins for hospitals due to the high baseline costs of the stent-grafts, equipment, and prolonged admission times [63]. Furthermore, prospective data indicate that the direct access-related costs of successful percutaneous procedures are frequently lower than surgical cutdowns (EUR 559 versus EUR 674 on average), a financial benefit primarily driven by the time saved in the operating theatre [43].

### 3.5. Quality of Life and Anaesthetic Considerations

Historically, the EVAR literature has focused heavily on survival and complication metrics, sometimes underreporting patient-centred outcomes. However, minimising surgical trauma translates directly to a better quality of life. By avoiding a groin incision, pEVAR patients require fewer opioids, experience reduced postoperative pain, and achieve faster functional recovery [21,64]. While pEVAR does not require it, patients should still be appropriately counselled on potential minor access site issues that can impact short-term comfort. For instance, a recent comparative analysis found that minor bleeding manageable with simple manual compression was significantly more frequent in the pEVAR group (6.8% versus 0.5% in the cutdown group), although the overall minor complication rates remained statistically equivalent between the two techniques [65].

Despite these minor bleeding events, the overarching avoidance of major surgical trauma may translate to improved health-related quality of life. Patients undergoing minimally invasive endovascular repairs report better early physical recovery, as measured by standardised tools such as the SF-36 physical component summary, compared to those subjected to open surgical exposures [66]. Importantly, the available literature comprehensively evaluating these patient-reported metrics is currently sparse and restricted to a small number of trials.

A clinical advantage of pEVAR is the freedom to perform the procedure without general anaesthesia. Because the trauma of a needle puncture is minimal compared to a surgical cutdown, reliable local or regional anaesthesia becomes entirely feasible. Despite this, local anaesthesia is utilised very rarely—a substantially underutilised opportunity for a patient demographic characterised by prevalent cardiopulmonary comorbidities [67,68]. Select outpatient programmes have successfully combined bilateral ilioinguinal nerve blocks with moderate IV sedation, achieving adequate comfort for less than USD 10 per case in pharmacological costs, with zero conversions to general anaesthesia; however, the generalisability of this approach remains to be established in larger, multicentre cohorts [69].

In the context of a ruptured AAA, avoiding general anaesthesia is associated with lower mortality rates. Inducing general anaesthesia in a bleeding, haemodynamically fragile patient precipitates acute haemodynamic collapse due to the loss of sympathetic vascular tone. Propensity-matched data show that using locoregional rather than general anaesthesia during ruptured AAA repair is associated with nearly halved 30-day mortality (14.6% vs. 29.2%) in a propensity-matched retrospective analysis; however, prospective randomised evidence confirming this mortality benefit is currently lacking [47]. Because locoregional anaesthesia is vastly easier to manage via percutaneous access than an open cutdown, prioritising pEVAR in emergency settings correlates with improved 30-day survival metrics. Finally, long-term morphological follow-ups spanning up to four years provide excellent reassurance that percutaneous closure devices do not cause delayed vascular injury or strictures, supporting the long-term safety profile of the pEVAR approach [70].

### 3.6. Summary of Key Quality-of-Life Studies

The summary of key quality-of-life studies is shown in Table 3.

## 4. Discussion

The transition from open surgical exposure to pEVAR represents a substantial methodological development in endovascular access. While the initial advent of EVAR advanced aortic repair by replacing laparotomy with transluminal access [5,6,8], pEVAR further refines this minimally invasive evolution by eliminating the surgical groin cutdown [16,17,18]. Synthesis of contemporary clinical evidence indicates that pEVAR has evolved beyond its experimental origins and represents a well-established alternative that may be considered preferable in anatomically suitable candidates, offering perioperative advantages that may extend beyond the access site [20,21,22,23,24].

While dual-suture devices remain the baseline for this technique, the field is rapidly diversifying. Furthermore, these methodological refinements have aligned with developments in the broader endovascular field; even as complex branched and fenestrated repairs demand increasingly large-calibre sheaths, contemporary percutaneous closure techniques have demonstrated sufficient efficacy to accommodate them without compromising success rates [34,35].

While current closure devices have comparable efficacy in achieving haemostasis, their distinct deployment mechanisms necessitate specific technical considerations to minimise access-site complications [30]. The aggregated clinical evidence substantially addresses prior concerns regarding the safety of percutaneous closure; however, a strict methodological distinction must be drawn when interpreting these outcomes. Conclusions asserting reduced operative duration, diminished early postoperative pain, and accelerated functional recovery are firmly grounded in robust RCTs. Conversely, evidence supporting specific anatomical risk factors, rates of late pseudoaneurysm formation, and overarching cost-effectiveness frameworks stems predominantly from retrospective, observational cohorts, necessitating cautious interpretation due to inherent selection biases. This caution is warranted because retrospective studies are prone to selection and reporting biases, whereas single-centre data lack the external validity required to generalise findings across broader clinical settings.

Comparative trials and extensive real-world registries note that pEVAR is clinically non-inferior to surgical cutdowns regarding primary aneurysm exclusion and overall survival [20,45,46]. However, its clinical utility is evidenced by decreased access-site morbidity. By avoiding formal surgical incisions, pEVAR reduces the incidence of traditional groin cutdown complications, including lymphatic leaks, surgical site infections, and nerve injuries, that historically prolonged hospitalisations and contributed to readmissions [12,13,44]. Easily managed access-site bleeding may occur slightly more frequently with percutaneous access and, while minor, it does represent a complication that should be addressed during preoperative planning [65]. These clinical findings are further supported by meta-analytic data showing that while pEVAR may reduce groin infections, lymphoceles, procedure time, and hospital stay, it does not eliminate local access-site complications such as haematoma or pseudoaneurysm, necessitating a more balanced clinical perspective rather than assuming unreserved superiority over open cutdown [71].

**Figure 4:**
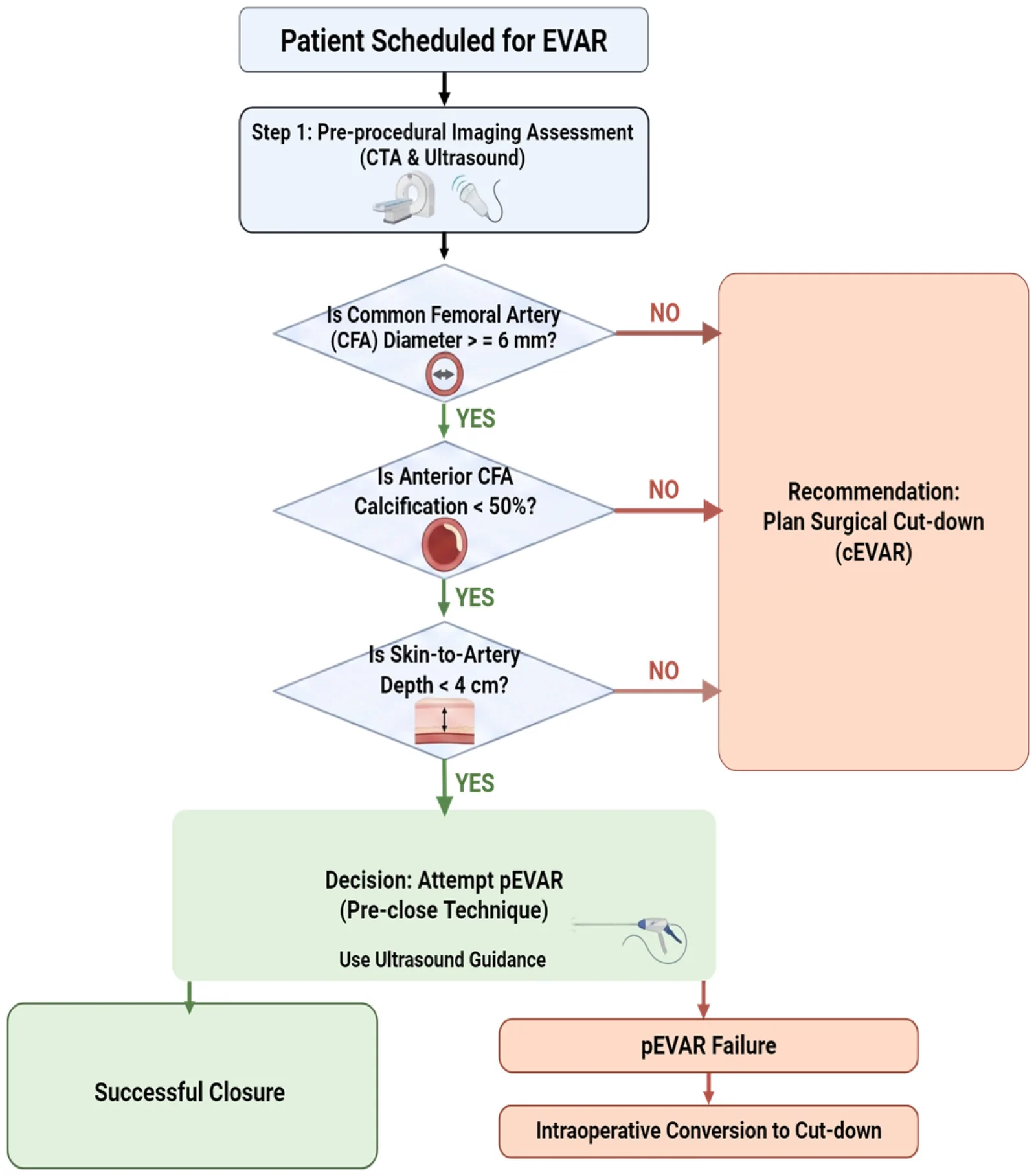
Workflow for patient management in the pEVAR procedure. Created in BioRender. Rodziewicz, B. (2026) https://biorender.com/m42gwi2 (accessed on 3 August 2026).

Contemporary meta-analyses examining percutaneous access in large-bore endovascular aortic interventions confirm operational advantages, including reduced operative duration, shorter hospital stays, and lower groin infection rates, yet highlight an increased risk of suture closure failure and pseudoaneurysm formation relative to surgical cutdown [72]. Nevertheless, the literature underscores that pEVAR is not universally applicable. Its success is intrinsically linked to strategic, imaging-driven patient selection. Preoperative computed tomography serves as an essential guide for access viability, allowing operators to systematically screen for unfavourable anatomies and mitigate technical failures (as shown in Figure 4) [24,45,49,50,51].

However, candidate selection in contemporary aortic interventions requires a broader multidisciplinary evaluation that integrates clinical frailty, comorbidity burden, underlying connective tissue disorders, procedural urgency, specific device profiles, institutional volume, and surveillance feasibility into individualised treatment planning [2].

Furthermore, the evidence highlights a significant learning curve. The technique demands a high degree of precision and ultrasound proficiency, confirming that institutional volume and operator experience are just as critical to closure success as the patient’s native anatomy [53]. Mitigating the pEVAR learning curve requires high institutional volume and mandatory ultrasound-guided arterial puncture [27,50,52]. Operators must simultaneously master bail-out strategies, such as rapid surgical conversion, to manage acute access-site failures [54,55].

pEVAR alters the perioperative pathway, facilitating protocols such as early ambulatory discharge. From an economic standpoint, the upfront cost of disposable closure devices is frequently offset by downstream savings, primarily driven by reduced operative durations, shorter hospital stays, and decreased intensive care utilisation, thereby mitigating the financial impact on hospital operating margins [22,57,62,63].

For the patient, avoiding surgical trauma directly translates to enhanced early postoperative well-being, characterised by reduced pain, lower opioid requirements, and an accelerated return to baseline functional independence [21,64,66].

Additionally, pEVAR facilitates locoregional anaesthetic pathways, which support quicker protocols in elective cases and offer a potential survival advantage in ruptured aneurysms by preserving sympathetic vascular tone [47,48,59,60,61,67]. Incorporating sex-aware imaging, individualised access planning along the entire iliofemoral axis, and maintaining a low threshold for planned surgical exposure or hybrid access strategies in hostile anatomies are possible steps to optimise outcomes and ensure patient safety across all demographics [55,56].

The substantial outcome heterogeneity across the literature is primarily driven by methodological divergence. For example, prospective trials use standardised endpoints, whereas retrospective analyses often group minor haematomas with major reinterventions under general “access-site morbidity”. Furthermore, varying follow-up durations complicate cross-study comparisons; short-term observations capture acute bleeding but may miss late consequences such as femoral stenosis. Thus, reported pEVAR outcomes depend on the interplay of patient selection, operator experience, closure devices, and study design.

Future pEVAR advancements rely on artificial intelligence to analyse CT scans for calcification and depth, automating patient selection. Additionally, physics-informed digital twins can simulate dynamic stent-graft interactions to predict seal failure and type IA endoleaks with high negative predictive value, enabling personalised repairs [73].

## 5. Limitations

A primary limitation of the current literature, and consequently this review, is the necessity to synthesise clinical outcomes across fundamentally different study designs. While early functional advantages of pEVAR are substantiated by evidence from RCTs, much of the nuanced clinical data, particularly concerning failure predictors, minor complications, and economic viability, relies on retrospective observational studies that are intrinsically susceptible to reporting and selection biases.

The current evidence base has several structural limitations. Most published data originate from centres with established pEVAR programmes and experienced operators, introducing selection bias at both the patient and institutional level; specialist-centre success rates consistently exceed national database analyses, reflecting the experience curve effect.

The current evidence is limited by substantial methodological heterogeneity among published studies, primarily concerning inconsistent complication definitions. Furthermore, the lack of long-term prospective comparative data impedes robust cross-study evaluation and leaves late vascular consequences incompletely characterised. Women remain substantially underrepresented despite having anatomical profiles that systematically increase pEVAR failure risk, limiting the generalisability of high success rates derived from male-predominant series.

Economic analyses are similarly limited, as they predominantly rely on data from a restricted number of healthcare systems, reducing their broader generalisability. Moreover, the unit costs of VCDs are usually a matter of non-public agreements between the manufacturers and facilities, and so data on them might be different depending on the region and timeframe in which the agreement was made.

Finally, formal methodological risk of bias cannot be ascertained because of the narrative form of this review.

## 6. Conclusions

Percutaneous EVAR (pEVAR) represents a viable alternative access strategy within contemporary vascular surgery. Although advancements in closure devices and “preclose” techniques show considerable promise, the safety profile of pEVAR, when compared to standard open femoral access, continues to require careful evaluation. While the technique offers potential advantages in reducing specific procedural morbidities, these benefits are strictly contingent upon rigorous adherence to anatomical selection criteria and substantial operator expertise. For carefully selected patients, pEVAR may facilitate postoperative recovery and provide theoretical benefits regarding healthcare resource utilisation. Nevertheless, further robust, long-term data are necessary to definitively validate these patient-centred outcomes and establish its broader applicability.

## Figures and Tables

**Table 1:** Data comparing Proglide and MANTA vascular closure devices [31].

Clinical Metric	Proglide	MANTA
Total Access sites evaluated	243 groins	127 groins
Anterior Calcification Present	14.8%	18.8%
Posterior Calcification Present	62.5%	66.9%
Successful Haemostasis	91.8%	90.5%
Rescue Operation Required	8.2%	9.1%
Required Additional Measures *	44.8%	21.2%
Secondary VCD for Initial Failure	4.5%	1.5%
Overall Pseudoaneurysm Rate	5.6%	4.0%

* Achieving haemostasis required adjuncts, such as an additional VCD, pressure dressing, Safeguard, or PTFE Felt Pledgets. VCD—vascular closure device.

**Table 2:** Most commonly used vascular closure devices in percutaneous endovascular aortic repair, with approximate unit cost *, mechanism, applicable sheath size, and technique, according to the manufacturer’s information.

VCD	Approximate Unit Cost	Mechanism	Sheath Size	Technique
Perclose ProGlide [36]	~USD 295 per device [37]	Suture-mediated	5–21 Fr.	Preclose 2 devices per site
MANTA [38]	Not publicly listed	Collagen plug + anchor	12–25 Fr.	Post-close 1 device per site
Prostar XL [39]	~USD 495 per device [40]	Suture-mediated	8.5–24 Fr.	Preclose 1 device per site
Perclose ProStyle [41]	Not publicly listed	Suture-mediated	5–21 Fr.	Preclose 2 devices per site

* Device costs are approximate baseline estimates that vary according to geographic region, local healthcare systems, and institutional purchasing agreements. Fr—French; VCD—vascular closure device.

**Table 3:** Summary of randomised and retrospective studies providing patient-centred outcome data after pEVAR, including health-state utility, functional recovery, pain, and anaesthetic effect on mortality.

Study	Design and Population	Quality-of-Life Key Findings
Nelson et al., 2014 (PEVAR Trial) [20]	RCT; 151 patients	Beneficial trends in groin pain and overall QoL in ProGlide group at 6 months.
Zhou et al., 2025 [21] (SWEET-EVAR)	RCT; 120 patients	EQ-5D-3L utility at 2 weeks: 0.876 (pEVAR) vs. 0.782 (cutdown). Return to normal activity: 16 vs. 28 days (*p* = 0.025). Pain duration: 4 vs. 8 days.
Uhlmann et al., 2018 [43]	RCT; 50 patients	No access-related complications in the percutaneous group; 8 complications in cEVAR patients.
Van Orden et al., 2018 [67]	Retrospective observational study; 8141 percutaneous EVARs	Local anaesthesia (used in only 9.3% of cases) is associated with fewer pulmonary complications and no increase in cardiac events or mortality.
Bennett et al., 2019 [47]	Retrospective observational study; 132 locoregional vs. 130 general anaesthesia	30-day mortality: 14.6% (locoregional) vs. 29.2% (general anaesthesia).
Phair et al., 2020 [64]	Retrospective observational study; 28 open repairs vs. 100 EVAR	Lower inpatient opioid consumption and lower pain scores at discharge in pEVAR patients compared to open repair.

EQ-5D, EuroQol five-dimension questionnaire; EVAR, endovascular abdominal aortic repair; cEVAR, cutdown EVAR; pEVAR, percutaneous EVAR; QoL, quality of life; RCT, randomised controlled trial.

## Data Availability

No new data were created or analyzed in this study. Data sharing is not applicable to this article.

## Supplementary Materials

The following supporting information can be downloaded at: https://www.mdpi.com/article/10.3390/jcm15166381/s1, File S1: Full search strings for each database; Table S1: Summary of all studies and records included in the review.

## Abbreviations

The following abbreviations are used in this manuscript:

AAA	Abdominal Aortic Aneurysm
pEVAR	Percutaneous Endovascular Aortic Aneurysm Repair
EVAR	Endovascular Aortic Aneurysm Repair
cEVAR	Cutdown Endovascular Aortic Aneurysm Repair
RCT	Randomised Controlled Trial
CFA	Common Femoral Artery
VCD	Vascular Closure Device
Fr	French
CT	Computed Tomography
QoL	Quality of Life
SF-36	36-Item Short Form Survey
EQ-5D	EuroQol Five-Dimension Questionnaire

## References

[1] Kent K.C., Zwolak R.M., Egorova N.N., Riles T.S., Manganaro A., Moskowitz A.J., Gelijns A.C., Greco G. (2010). Analysis of risk factors for abdominal aortic aneurysm in a cohort of more than 3 million individuals. J. Vasc. Surg..

[2] Wanhainen A., Van Herzeele I., Bastos Goncalves F., Montoya S.B., Berard X., Boyle J.R., D’oRia M., Prendes C.F., Karkos C.D., Kazimierczak A. (2024). European Society for Vascular Surgery (ESVS) 2024 Clinical Practice Guidelines on the Management of Abdominal Aorto-iliac Artery Aneurysms. Eur. J. Vasc. Endovasc. Surg..

[3] Lederle F.A., Johnson G.R., Wilson S.E., Ballard D.J., Jordan J.W.D., Blebea J., Littooy F.N., Freischlag J.A., Bandyk D., Rapp J.H. (2002). Rupture Rate of Large Abdominal Aortic Aneurysms in Patients Refusing or Unfit for Elective Repair. JAMA.

[4] Powell J.T., Sweeting M.J., Ulug P., Blankensteijn J.D., A Lederle F., Becquemin J.-P., Greenhalgh R.M., Beard J.D., Buxton M.J., Brown L.C. (2017). Meta-analysis of individual-patient data from EVAR-1, DREAM, OVER and ACE trials comparing outcomes of endovascular or open repair for abdominal aortic aneurysm over 5 years. Br. J. Surg..

[5] Criado F.J. (2012). Nicholay Volodos and the Origins of Endovascular Grafts. J. Endovasc. Ther..

[6] Ivancev K., Vogelzang R. (2020). A 35 Year History of Stent Grafting, and How EVAR Conquered the World. Eur. J. Vasc. Endovasc. Surg..

[7] EVAR trial participants (2005). Endovascular aneurysm repair versus open repair in patients with abdominal aortic aneurysm (EVAR trial 1): Randomised controlled trial. Lancet.

[8] Parodi J.C., Palmaz J.C., Barone H.D. (1991). Transfemoral Intraluminal Graft Implantation for Abdominal Aortic Aneurysms. Ann. Vasc. Surg..

[9] Blankensteijn J.D., de Jong S.E., Prinssen M., van der Ham A.C., Buth J., van Sterkenburg S.M., Verhagen H.J., Buskens E., Grobbee D.E. (2005). Two-year Outcomes after Conventional or Endovascular Repair of Abdominal Aortic Aneurysms. N. Engl. J. Med..

[10] Lederle F.A., Freischlag J.A., Kyriakides T.C., Padberg F.T., Matsumura J.S., Kohler T.R., Lin P.H., Jean-Claude J.M., Cikrit D.F., Swanson K.M. (2009). Outcomes Following Endovascular vs. Open Repair of Abdominal Aortic Aneurysm: A randomized trial. JAMA.

[11] Becquemin J.P., Pillet J.C., Lescalie F., Sapoval M., Goueffic Y., Lermusiaux P., Steinmetz E., Marzelle J., Trialists A. (2011). A randomized controlled trial of endovascular aneurysm repair versus open surgery for abdominal aortic aneurysm in low- to moderate-risk patients. J. Vasc. Surg..

[12] Vatakencherry G., Molloy C., Sheth N., Liao M., Lam C.K. (2018). Percutaneous access planning, techniques and considerations for endovascular aortic repair (EVAR). Cardiovasc. Diagn. Ther..

[13] Torsello G., Kasprzak B., Klenk E., Tessarek J., Osada N., Torsello G.F. (2003). Endovascular suture versus cutdown for endovascular aneurysm repair: A prospective randomized pilot study. J. Vasc. Surg..

[14] Sakalihasan N., Michel J.B., Katsargyris A., Kuivaniemi H., Defraigne J.-O., Nchimi A., Powell J.T., Yoshimura K., Hultgren R. (2018). Abdominal aortic aneurysms. Nat. Rev. Dis. Primers.

[15] Banceu C.M., Gurzu S., Satala C.-B., Ghiga D., Neamtu M.H., Voth V., Liebrich M., Suciu H. (2023). Histopathological Gap in Aortic Diseases: A Prospective Analysis. Int. J. Mol. Sci..

[16] Szpotan T., Czernik M., Majos A. (2023). Suture-mediated closure devices for percutaneous endovascular abdominal aneurysm repair. Pol. J. Radiol..

[17] Howell M., Dougherty K., Strickman N., Krajcer Z. (2002). Percutaneous repair of abdominal aortic aneurysm using the AneuRx stent graft and the percutaneous vascular surgery device. Catheter. Cardiovasc. Interv..

[18] Georgiadis G.S., Antoniou G.A., Papaioakim M., Georgakarakos E., Trellopoulos G., Papanas N., Lazarides M.K. (2011). A Meta-Analysis of Outcome after Percutaneous Endovascular Aortic Aneurysm Repair using Different Size Sheaths. J. Endovasc. Ther..

[19] Malkawi A.H., Hinchliffe R.J., Holt P.J., Loftus I.M., Thompson M.M. (2010). Percutaneous Access for Endovascular Aneurysm Repair: A Systematic Review. Eur. J. Vasc. Endovasc. Surg..

[20] Nelson P.R., Krajcer Z., Kansal N., Rao V., Bianchi C., Hashemi H., Jones P., Bacharach J.M. (2014). A multicenter, randomized, controlled trial of totally percutaneous access versus open femoral exposure for EVAR (the PEVAR trial). J. Vasc. Surg..

[21] Zhou Y., Wang J., Zhao J., Yuan D., Weng C., Huang B., Wang T. (2025). Comparison of percutaneous versus cutdown access in patients after endovascular abdominal aortic repair: A randomized controlled trial (SWEET-EVAR trial). Int. J. Surg..

[22] Hong J.C., Yang G.K., Delarmente B.A., Khera R., Price J., Faulds J., Chen J.C. (2020). Cost-minimization study of the percutaneous approach to endovascular aortic aneurysm repair. J. Vasc. Surg..

[23] O’Brien-Irr M.S., Harris L.M., Dosluoglu H.H., Cherr G.S., Rivero M., Noor S., Curl G.R., Dryjski M.L. (2017). Factors that affect cost and clinical outcome of endovascular aortic repair for abdominal aortic aneurysm. J. Vasc. Surg..

[24] Eisenack M., Umscheid T., Tessarek J., Torsello G.F., Torsello G.B. (2009). Percutaneous Endovascular Aortic Aneurysm Repair: A Prospective Evaluation of Safety, Efficiency, and Risk Factors. J. Endovasc. Ther..

[25] Sukhera K. (2022). Narrative Reviews: Flexible, Rigorous, and Practical. J. Grad. Med. Educ..

[26] Page M.J., McKenzie J.E., Bossuyt P.M., Boutron I., Hoffmann T.C., Mulrow C.D., Shamseer L., Tetzlaff J.M., Akl E.A., Brennan S.E. (2021). The PRISMA 2020 statement: An updated guideline for reporting systematic reviews. BMJ.

[27] Jaffan A.A.A., Prince E.A., Hampson C.O., Murphy T.P. (2013). The Preclose Technique in Percutaneous Endovascular Aortic Repair: A Systematic Literature Review and Meta-analysis. J. Cardiovasc. Interv. Radiol..

[28] Oddi F.M., Fresilli M., Pennetta F.F., Micali R., Marchetti A.A., Ippoliti A. (2025). Ultrasound-guided Femoseal^®^ vascular closure device in antegrade common femoral artery puncture. J. Vasc. Access.

[29] Michiels J., Peeters M., Uittenbogaart M., Oosterveld R., Bloo L., Loos M., Bouwman L., Janssen R., Elshof J.-W., Yazar O. (2025). Experience with a Large-Bore Vascular Closure Device in Patients Undergoing Percutaneous Endovascular Aneurysm Repair (EVAR): A Multicentre Study. Cardiovasc. Interv. Radiol..

[30] Hakeem A., Najem M., Khokher Z., Chaudhuri A. (2023). A Comparative Analysis of the Early and Late Complication Rates and the Effect of Calcification on the Efficacy of MANTA and ProGlide Vascular Closure Devices. Cureus.

[31] Noori V.J., Eldrup-Jørgensen J. (2018). A systematic review of vascular closure devices for femoral artery puncture sites. J. Vasc. Surg..

[32] Montán C., Lehti L., Holst J., Björses K., Resch T.A. (2011). Short- and Midterm Results of the Fascia Suture Technique for Closure of Femoral Artery Access Sites After Endovascular Aneurysm Repair. J. Endovasc. Ther..

[33] Harrison G.J., Thavarajan D., Brennan J.A., Vallabhaneni S.R., McWilliams R.G., Fisher R.K. (2010). Fascial Closure Following Percutaneous Endovascular Aneurysm Repair. Eur. J. Vasc. Endovasc. Surg..

[34] Melloni A., Grandi A., Spelta S., Salvati S., Loschi D., Lembo R., Melissano G., Chiesa R., Bertoglio L. (2020). Outcomes of routine use of percutaneous access with large-bore introducer sheaths (>21F Outer Diameter) during endovascular aneurysm repair. J. Vasc. Surg..

[35] de Souza L.R., Oderich G.S., Banga P.V., Hofer J.M., Wigham J.R., Cha S., Gloviczki P. (2015). Outcomes of total percutaneous endovascular aortic repair for thoracic, fenestrated, and branched endografts. J. Vasc. Surg..

[36] Overview of Perclose ProGlide|Abbott. https://www.cardiovascular.abbott/us/en/hcp/products/peripheral-intervention/vessel-closure/perclose-proglide-suture-mediated-closure-system/overview.html.

[37] Lee W.A., Brown M.P., Nelson P.R., Huber T.S. (2007). Total percutaneous access for endovascular aortic aneurysm repair (“Preclose” technique). J. Vasc. Surg..

[38] MANTA^®^ Vascular Closure Device|US|Teleflex. https://teleflex.com/usa/en/product-areas/interventional/vascular-access-closure/manta-vascular-closure-device/index.html.

[39] Prostar XL Percutaneous Vascular Surgical System|Abbott. https://www.cardiovascular.abbott/us/en/hcp/products/peripheral-intervention/vessel-closure/prostar-xl-percutaneous-vascular-surgical-system.html.

[40] Lee W.A., Moore W.S., Ahn S.S. (2011). Arterial Closure Devices. Endovascular Surgery.

[41] Perclose ProStyle Suture-Mediated Closure System Overview|Abbott. https://www.cardiovascular.abbott/int/en/hcp/products/peripheral-intervention/vessel-closure/perclose-prostyle-suture-mediated-closure-system/overview.html.

[42] Maniotis C., Andreou C., Karalis I., Koutouzi G., Agelaki M., Koutouzis M. (2017). A systematic review on the safety of Prostar XL versus ProGlide after TAVR and EVAR. Cardiovasc. Revasc. Med..

[43] Uhlmann M.E., Walter C., Taher F., Plimon M., Falkensammer J., Assadian A. (2018). Successful percutaneous access for endovascular aneurysm repair is significantly cheaper than femoral cutdown in a prospective randomized trial. J. Vasc. Surg..

[44] Read M., Nguyen T., Swan K., Arnaoutakis D.J., Dua A., Toloza E., Shames M., Bailey C., Latz C.A. (2024). Cutdown is Associated with Higher 30-day Unplanned Readmissions and Wound Complications than Percutaneous Access for EVAR. Ann. Vasc. Surg..

[45] Pratesi G., Barbante M., Pulli R., Fargion A., Dorigo W., Bisceglie R., Ippoliti A., Pratesi C., IPER Registry Collaborators (2015). Italian Percutaneous EVAR (IPER) Registry: Outcomes of 2381 percutaneous femoral access sites’ closure. J. Cardiovasc. Surg..

[46] Melani C., Bastianon M., Mozzetta G., Mozzetta G., DI Gregorio S., DI Bartolo M., Capone A., Pratesi C., Pulli R., Mauri F. (2022). Multicenter real-life study on access-related outcomes after EVAR: Percutaneous is the way. Ital. J. Vasc. Endovasc. Surg..

[47] Bennett K.M., McAninch C.M., Scarborough J.E. (2019). Locoregional anesthesia is associated with lower 30-day mortality than general anesthesia in patients undergoing endovascular repair of ruptured abdominal aortic aneurysm. J. Vasc. Surg..

[48] Tay S., Zaghloul M.S., Shafqat M., Yang C., Desai K.A., De Silva G., Sanchez L.A., Zayed M.A. (2022). Totally percutaneous endovascular repair for ruptured abdominal aortic aneurysms. Front. Surg..

[49] Manunga J.M., Gloviczki P., Oderich G.S., Kalra M., Duncan A.A., Fleming M.D., Bower T.C. (2013). Femoral artery calcification as a determinant of success for percutaneous access for endovascular abdominal aortic aneurysm repair. J. Vasc. Surg..

[50] Gradinariu G., Lyons O., Musajee M., Yap T., Johnson O., Bujoreanu I., Shalhoub J., Wilkins J., Gkoutzios P., Tyrrell M. (2022). Predictors of percutaneous access-related complications in aortic endovascular procedures: ‘real-world’ insights and a comparison to open access. Int. Angiol..

[51] Bensley R.P., Hurks R., Huang Z., Pomposelli F., Hamdan A., Wyers M., Chaikof E., Schermerhorn M.L. (2012). Ultrasound-guided percutaneous endovascular aneurysm repair success is predicted by access vessel diameter. J. Vasc. Surg..

[52] Ashrafi M., Al-Jarrah Q., Anandarajah M., Ashleigh R., Welch M., Baguneid M. (2016). Single-Center Experience Following the Introduction of a Percutaneous Endovascular Aneurysm Repair First Approach. Angiology.

[53] Bechara C.F., Barshes N.R., Pisimisis G., Chen H., Pak T., Lin P.H., Kougias P. (2013). Predicting the learning curve and failures of total percutaneous endovascular aortic aneurysm repair. J. Vasc. Surg..

[54] Al-Khatib W.K., Zayed M.A., Harris E.J., Dalman R.L., Lee J.T. (2012). Selective Use of Percutaneous Endovascular Aneurysm Repair in Women Leads to Fewer Groin Complications. Ann. Vasc. Surg..

[55] Liu Y., Yang Y., Zhao J., Chen X., Wang J., Ma Y., Huang B., Yuan D., Du X. (2020). Systematic review and meta-analysis of sex differences in outcomes after endovascular aneurysm repair for infrarenal abdominal aortic aneurysm. J. Vasc. Surg..

[56] Marzano A., Flora F., Jabbour J., Martinelli O., Cuozzo S. (2026). Sex Differences in Outcomes After Endovascular Abdominal Aortic Aneurysm Repair: A Systematic Review and Narrative Synthesis. Rev. Cardiovasc. Med..

[57] Thurston J.S., Camara A., Alcasid N., White S.B., Patel P.J., Rossi P.J., Hieb R.E., Lee C.J. (2019). Outcomes and Cost Comparison of Percutaneous Endovascular Aortic Repair versus Endovascular Aortic Repair With Open Femoral Exposure. J. Surg. Res..

[58] Mathisen S.R., Berge S.T. (2026). A seven-year single-center experience with large-bore percutaneous closure in endovascular aneurysm repair. Vascular.

[59] Naiem A.A., Doonan R.J., Guigui A., Obrand D.I., Bayne J.P., MacKenzie K.S., Steinmetz O.K., Girsowicz E., Gill H.L. (2022). Feasibility and Cost Analysis of Ambulatory Endovascular Aneurysm Repair. J. Endovasc. Ther..

[60] Dosluoglu H.H., Lall P., Blochle R., Harris L.M., Dryjski M.L. (2014). Ambulatory percutaneous endovascular abdominal aortic aneurysm repair. J. Vasc. Surg..

[61] Kameda Y., Nemoto N., Inoue B., Takaesu S., Takenaka H., Nagashima Y., Anzai H. (2025). The practice of percutaneous EVAR under local anesthesia. Ann. Vasc. Dis..

[62] Altoijry A., Alsheikh S., Alanezi T., Aljabri B., Aldossary M.Y., Altuwaijri T., Iqbal K. (2023). Percutaneous versus Cutdown Access for Endovascular Aortic Repair. Heart Surg. Forum.

[63] Krajcer Z., Ramaiah V.G., Henao E.A., Nelson W.K., Moursi M.M., A Rajasinghe H., Anderson L.H., E Miller L. (2019). Comparison of perioperative costs with fast-track vs. standard endovascular aneurysm repair. Vasc. Health Risk Manag..

[64] Phair J., Carnevale M., Levine D., Lipsitz E.C., Scher L., Shariff S., Garg K. (2020). Underutilization of Nonopioid Pain Medication in Patients Undergoing Abdominal Aortic Aneurysm Repair. Ann. Vasc. Surg..

[65] Rebelo A., Voss P., Ronellenfitsch U., Sekulla C., Ukkat J. (2022). Comparison of percutaneous and cutdown access-related minor complications after endovascular aortic repair. Exp. Ther. Med..

[66] Ķīsis K., Krieviņš D., Naškoviča K., Gediņš M., Šavlovskis J., Ezīte N., Lietuvietis E., Zariņš K. (2012). Quality of Life After Endovascular Abdominal Aortic Aneurysm Repair: Nellix Sac-Anchoring Endoprosthesis Versus Open Surgery. Medicina.

[67] Van Orden K., Farber A., Schermerhorn M.L., Schermerhorn M.L., Goodney P.P., Kalish J.A., Jones D.W., Rybin D., Siracuse J.J. (2018). Local anesthesia for percutaneous endovascular abdominal aortic aneurysm repair is associated with fewer pulmonary complications. J. Vasc. Surg..

[68] Ioannou C.V., Kontopodis N., Kehagias E., Papaioannou A., Kafetzakis A., Papadopoulos G., Pantidis D., Tsetis D. (2015). Endovascular aneurysm repair with the Ovation TriVascular Stent Graft System utilizing a predominantly percutaneous approach under local anaesthesia. Br. J. Radiol..

[69] Harlin S.A., Grissom R.A., Lecroy C., Pouliot S.M., Harlin S.A. (2016). A novel anesthetic technique for PEVAR. Ann. Vasc. Surg..

[70] Lee W.A., Brown M.P., Nelson P.R., Huber T.S., Seeger J.M. (2008). Midterm outcomes of femoral arteries after percutaneous EVAR using the Preclose technique. J. Vasc. Surg..

[71] Hajibandeh S., Hajibandeh S., Antoniou S.A., Child E., Torella F., Antoniou G.A. (2016). Percutaneous access for endovascular aortic aneurysm repair: A systematic review and meta-analysis. Vascular.

[72] Liang Z., Huang Z., Wu M., Lin L., Chen B., Yin S., Li H., Quan J., Liang W., Huang X. (2025). Comparison of percutaneous vs. cutdown access for endovascular aortic repair in the treatment of type B aortic dissection: A meta-analysis. Front. Cardiovasc. Med..

[73] Cho S., Kim H., Joh J. (2026). Digital Twin and Artificial Intelligence Technologies to Assess the Type IA Endoleak. Bioengineering.

