# Supplementary material for: Present-Day State of Percutaneous Endovascular Aortic Aneurysm Repair
Source: J Clin Med. 2026 Aug 18;15(16):6381. doi: 10.3390/jcm15166381 (PMC13513119; doi:10.3390/jcm15166381)
Supplement: Supplementary file 1 [file jcm-15-06381-s001.zip › Supplementary Material File S1.pdf]

File S1: Full search strings for each database.

Pubmed

((("pEVAR"[tiab] OR "percutaneous endovascular aortic repair"[tiab] OR "percutaneous endovascular aneurysm repair"[tiab] OR "percutaneous EVAR"[tiab]) AND ("Aneurysm"[Mesh] OR "aneurysm"[tiab])) OR (("percutaneous access"[tiab] OR "totally percutaneous"[tiab] OR "femoral access"[tiab] OR "femoral cutdown"[tiab] OR "open femoral exposure"[tiab] OR "preclose"[tiab] OR "vascular closure device"[tiab] OR "vascular closure devices"[tiab] OR "ProGlide"[tiab] OR "Prostar"[tiab] OR "MANTA"[tiab]) AND ("EVAR"[ti] OR "endovascular aneurysm repair"[ti] OR "endovascular aortic repair"[ti]) AND ("Aneurysm"[Mesh] OR "aneurysm"[tiab])))

Scopus

((TITLE-ABS-KEY ("pEVAR" OR "percutaneous endovascular aortic repair" OR "percutaneous endovascular aneurysm repair" OR "percutaneous EVAR") AND TITLE-ABS-KEY ("aneurysm")) OR (TITLE-ABS ("percutaneous access" OR "totally percutaneous" OR "femoral access" OR "femoral cutdown" OR "open femoral exposure" OR "preclose" OR "vascular closure device" OR "vascular closure devices" OR "ProGlide" OR "Prostar" OR "MANTA") AND TITLE ("EVAR" OR "endovascular aneurysm repair" OR "endovascular aortic repair") AND TITLE-ABS ("aneurysm")))

Web of Science

((TS=("pEVAR" OR "percutaneous endovascular aortic repair" OR "percutaneous endovascular aneurysm repair" OR "percutaneous EVAR") AND TS=("aneurysm")) OR ((AB=("percutaneous access" OR "totally percutaneous" OR "femoral access" OR "femoral cutdown" OR "open femoral exposure" OR "preclose" OR "vascular closure device" OR "vascular closure devices" OR "ProGlide" OR "Prostar" OR "MANTA") OR TI=("percutaneous access" OR "totally percutaneous" OR "femoral access" OR "femoral cutdown" OR "open femoral exposure" OR "preclose" OR "vascular closure device" OR "vascular closure devices" OR "ProGlide" OR "Prostar" OR "MANTA")) AND TI=("EVAR" OR "endovascular aneurysm repair" OR "endovascular aortic repair") AND (TI=("aneurysm") OR AB=("aneurysm")))))
