# Supplementary material for: Present-Day State of Percutaneous Endovascular Aortic Aneurysm Repair
Source: J Clin Med. 2026 Aug 18;15(16):6381. doi: 10.3390/jcm15166381 (PMC13513119; doi:10.3390/jcm15166381)
Supplement: Supplementary file 1 [file jcm-15-06381-s001.zip › Supplementary Material Table S1.pdf]

Table S1. Summary of all studies and records included in the review.

Study	Design	Population	Intervention	Outcome
Kent et al. (2010) [1]	Retrospective Observational Study	3.1 million patients who completed a medical and lifestyle questionnaire and were evaluated by ultrasound imaging for the presence of Abdominal Aortic Aneurysm (AAA).	Ultrasound screening and multivariable analysis of risk factors.	Identified a strong AAA association with years of smoking and excess weight. Created a predictive scoring system: estimated 1.1 million AAAs in the US, including 569,000 among women, nonsmokers, and those <65 years (who are often missed by current screening guidelines).
Wanhainen et al. (2024) [2]	Guidelines	Patients with abdominal aorto-iliac artery aneurysms.	Establishment of clinical management, diagnostic, and screening standards based on evidence and expert opinion.	Issued 160 recommendations graded by strength (classes I to III) and level of evidence (A to C).
Lederle et al. (2002) [3]	Prospective Observational Study	198 patients with AAA at least 5.5 cm in diameter for whom elective repair was not expected in the next 6 months because of medical contraindications to surgery or patient refusal.	Conservative management (observation without surgical repair).	1-year rupture incidence based on initial diameter: 5.5–5.9 cm: 9.4%; 6.0–6.9 cm: 10.2%; ≥7.0 cm: 32.5%. For AAAs reaching 8.0 cm, 25.7% ruptured within 6 months.

Powell et al. (2017) [4]	Meta-analysis	2,783 patients from EVAR-1, DREAM, ACE, and OVER trials (14,245 person-years, median follow-up 5.5 years).	Endovascular Aortic Repair (EVAR) compared to open surgical repair (OSR).	Early mortality (0–6 months) was lower for EVAR (HR 0.61; 30-day OR 0.40). Survival curves converged later. Beyond 3 years, aneurysm-related mortality was higher in the EVAR group (HR 5.16).
Criado F.J. (2012) [5]	Historical Review	-	-	Description of the origins of endovascular grafts.
Ivancev et al. (2020) [6]	Historical Review	-	-	Evolution of endovascular techniques over 35 years.
Greenhalgh et al. (2005) [7]	RCT (Randomised Control Trial)	1082 patients aged 60 years or older who had aneurysms of at least 5·5 cm in diameter and who had been referred to one of 34 hospitals proficient in the EVAR technique.	EVAR (n=543) compared to OSR (n=539).	At 4 years, all-cause mortality was similar (~28%). EVAR had lower aneurysm-related mortality (4% vs 7%) but a higher postoperative complication rate (41% vs 9%) and higher hospital costs (£13,257 vs £9,946).

Parodi et al. (1991) [8]	Animal experimentation and initial clinical trials	Animal models and 5 patients with serious comorbidities.	Transfemoral intraluminal placement of a stent-anchored Dacron prosthetic graft.	Achieved successful aneurysm exclusion by creating friction seals with balloon-expandable stents, proving the feasibility of this early technique.
Blankensteijn et al. (2005) [9]	RCT	351 patients who had received a diagnosis of abdominal aortic aneurysm of at least 5 cm in diameter and who were considered suitable candidates.	EVAR compared to OSR.	2-year cumulative survival rates were similar (OSR: 89.6% vs EVAR: 89.7%). Aneurysm-related mortality was 5.7% vs 2.1%, respectively, the advantage of EVAR was entirely accounted for by events in the perioperative period, with no significant difference in subsequent aneurysm-related mortality.
Lederle et al. (2009) [10]	RCT	881 patients (aged ≥49 years) from 42 Veterans Affairs Medical Centers with eligible AAA who were candidates for both elective EVAR and OSR of AAA.	EVAR (n=444) compared to OSR (n=437).	Perioperative mortality was lower for EVAR (0.5% vs 3.0%, p=0.004). This mortality difference was not sustained at 2 years (7.0% vs 9.8%; p=0.13). EVAR reduced procedure time, blood loss, and hospital/ICU stay.

Becquemin et al. (2011) [11]	RCT	316 patients with >5 cm aneurysms were randomized in institutions with proven expertise for EVAR and OSR.	EVAR (n=150) compared to OSR (n=149).	At 3 years, survival free of death/major events was similar (OSR 85.1% vs EVAR 82.4%). However, EVAR had a higher reintervention rate (16% vs 2.4%, P<0.0001) and a trend toward higher aneurysm-related mortality (4% vs 0.7%). Conclusion: OSR is as safe and more durable.
Vatakencherry et al. (2018) [12]	Technical review	Patients undergoing percutaneous EVAR (pEVAR).	pEVAR techniques, focusing on the "preclose technique," ultrasound guidance, and alternative access routes (brachial, radial, transcaval, transapical, conduits).	Outlines that preclosure with two Perclose Proglide devices yields shorter procedure times and fewer complications than cutdown. Discusses bailout strategies and novel access options for hostile anatomy.
Torsello et al. (2003) [13]	RCT	30 patients (accounting for 55 femoral arteries cannulated with large-bore 14F-25F introducers) undergoing EVAR.	pEVAR technique using endovascular suture devices (Group A) compared to femoral cutdown EVAR (cEVAR)(Group B).	pEVAR access reduced mean surgery time (86.7 vs 107.8 minutes, $P < 0.05$) and time to ambulation (20.1 vs 33.1 hours, $P < 0.001$). Complication rates were similar, though the percutaneous method cost an average of 99.2 € more due to the device cost.

Sakalihasan et al. (2018) [14]	Narrative Review	Patients with or at risk for AAA.	General overview of screening, functional imaging, and elective repair (EVAR or OSR).	Emphasizes that AAA rupture carries a 65–85% mortality rate. Identifies smoking, male sex, and family history as major risk factors, noting that both OSR and EVAR carry non-negligible morbidity/mortality.
Banceu et al. (2023) [15]	Prospective histopathological study	108 adult patients who received aortac replacement for Aortic Dissection (AD) or Aortic Aneurysm (AA) between 2019–2021.	Histological assessment and scoring of the aortic wall (medial degeneration, MEMA, elastic fragmentation) from surgical samples.	AD was associated with a 3.857 times higher risk of in-hospital mortality and 4.188 times higher risk of overall mortality compared to AA. Each unit increase in the histopathological score increased the odds of in-hospital mortality by 3.073.
Szpotan et al. (2023) [16]	Retrospective observational study	202 patients with AAA qualified for pEVAR with Perclose Proglide preclosure technique.	pEVAR preclosure technique using Perclose Proglide with large-bore sheaths (12F to 22F).	Large-bore device pEVAR procedures with closure devices are effective and safe. High effectiveness of haemostasis (98%) was observed in the study group, with a low percentage of complications (2.6%). There was no correlation between size of the vascular access and the lack of haemostasis found in the study.

Howell et al. (2002) [17]	Prospective clinical study	30 patients underwent an attempted pEVAR. These patients were followed prospectively to assess the success of the procedure.	pEVAR using the AneuRx stent graft and the Prostar XL device (preclose technique).	93% (28/30) had successful pEVAR of both common femoral artery (CFA) access sites. The success rate for larger 22 Fr sheaths was 96%. There were zero in-hospital groin complications.
Georgiadis et al. (2011) [18]	Meta-analysis	17 studies (1 RCT, 16 observational) including 1,440 patients and 2,447 femoral access sites.	pEVAR using suture-mediated closure devices, comparing smaller sheaths (≤18-F) vs. larger sheaths (≥20-F).	Primary success rates were better with ≤18-F sheaths than with ≥20-F sheaths (OR 1.78, p=0.002). Larger-bore sheaths (≥20-F) were identified as predictors of primary closure failure requiring conversion to cutdown.
Malkawi et al. (2010) [19]	Systematic Review	22 papers attempting pEVAR across 1,087 patients (1,751 groins).	pEVAR, pEVAR vs cEVAR.	The overall success rate of percutaneous arterial closure was 92%, with a access related complication rate of 4.4%. pEVAR was associated with fewer access-related complications (RR 0.47) and reduced operative time compared to cEVAR.

Nelson et al. (2014) [20]	RCT	151 patients undergoing EVAR.	pEVAR using 8F Perclose ProGlide (n=50) or 10F Prostar XL (n=51) compared to cEVAR (n=50).	1-month primary treatment success was 88% for ProGlide, 78% for Prostar, and 78% for cEVAR. ProGlide demonstrated statistical non-inferiority to cEVAR. Both percutaneous devices yielded significantly shorter times to haemostasis and procedure completion compared to cEVAR.
Zhou et al. (2025) [21]	RCT	120 patients (240 access sites) diagnosed with abdominal aortic diseases.	pEVAR vs cEVAR.	pEVAR was non-inferior for complications (8.1% vs 14.7%). It was superior for patient-centred outcomes, including faster return to normal life (16 vs 28 days), shorter pain duration (4 vs 8 days), and decreased analgesic use.
Hong et al. (2020) [22]	Cost-minimization study (decision tree modelling)	6,876 EVARs identified from the 2014 National Inpatient Sample database.	pEVAR vs cEVAR.	pEVAR resulted in a mean cost savings of $751 per procedure ($1,287 vs $2,038). The savings were primarily driven by a shorter length of hospitalization (-1.4 days). pEVAR remained cost-saving in 82.6% of modelled simulations.

O'Brien-Irr et al. (2017) [23]	Retrospective clinical and financial analysis	67 patients undergoing elective EVAR (completed between January 2012 and June 2013).	EVAR using different graft types (Graft A vs. Graft B), adjunctive components, and attempt at pEVAR.	Use of additional cuffs/extensions increased total costs by 20% and graft costs by 36%. Bilateral pEVAR (successful in 73%) was associated with shorter operating time, lower OR/hospital costs, and higher likelihood of discharge on postoperative day 1 compared to unilateral or failed pEVAR.
Eisenack et al. (2009) [24]	Prospective observational study	500 patients (417 men) treated for AAs with large sheaths (14-F to 24-F).	pEVAR using a single Prostar XL 10-F device with the preclose technique.	Primary success was achieved in 96.1% of pEVAR. Early conversion to cEVAR strongly correlated with CFA calcification (OR 74.5) and operator experience (OR 43.2), but not obesity. A groin scar was a significant risk factor for late access site repairs (OR 48.8).
Sukhera K. (2022) [25]	Methodological article	-	-	Explains that narrative reviews align with subjectivist/interpretivist paradigms, contrasting with systematic reviews. They are valuable for exploring complex, broad, or under-researched topics by providing synthesis,

				interpretation, and critique from diverse perspectives.
Page et al. (2021) [26]	Methodological article	-	-	Introduces a 27-item checklist and updated flow diagrams that reflect advances in methods (e.g., machine learning, risk of bias assessment, certainty of evidence) to ensure transparent, complete, and accurate reporting of systematic reviews.
Jaffan et al. (2013) [27]	Systematic review and meta-analysis	36 articles comprising 2,257 patients and 3,606 arterial accesses (published 1999–2012).	pEVAR using the preclose technique.	The technical success rate per arterial access was 94%, with a groin complication rate of 3.6% (only 1.6% required OSR). Ultrasound guidance significantly increased technical success, while larger sheath sizes (≥20F) significantly increased the technical failure rate.
Oddi et al. (2025) [28]	Retrospective observational study	301 patients undergoing percutaneous lower limb revascularization using 6- to 8-Fr sheaths.	Haemostasis using the ultrasound-guided FemoSeal vascular closure device (VCD) in antegrade CFA puncture.	Technical success was achieved in 99%. Complications included three pseudoaneurysms (one treated with thrombin injection, two managed

				during follow-up) and two conversions to manual compression. Conclusion: Safe and effective for achieving haemostasis.
Michiels et al. (2025) [29]	Retrospective observational study	549 patients (including 140 classified as obese) undergoing pEVAR (1,024 CFAs).	CFA closure using the MANTA large-bore VCD.	Overall technical success was 98% with a 3.1% complication rate. In obese patients (BMI ≥30), technical success was 98.9% with a 1.5% complication rate. The MANTA device is effective and safe, showing comparable, highly favourable outcomes in both obese and non-obese patients.
Hakeem et al. (2023) [30]	Retrospective observational study	370 large-bore CFA accesses in 205 endovascular procedures (predominantly EVAR).	Closure with Perclose ProGlide (Group A, 64.9%) compared to MANTA (Group B, 35.1%).	Successful haemostasis (including use of adjuncts) was similar (91.8% ProGlide vs 90.5% MANTA). ProGlide required more adjuncts. Arterial calcification site (anterior vs. posterior) did not significantly impact haemostasis success for either device.

Noori et al. (2018) [31]	Systematic review	34 articles (clinical trials within the last 10 years).	Use of FDA-approved (VCDs) compared to Manual Compression (MC).	VCDs improved patient comfort, time to haemostasis, and ambulation. However, overall complication rates, safety, and efficacy remained comparable (12% VCDs vs 13% MC). VCDs had a slightly increased risk of infectious (0.6% vs 0.2%) and thrombotic complications (0.3% vs 0%) compared to MC.
Montán et al. (2011) [32]	Retrospective observational study	100 EVAR cases (187 femoral access sites, 160 closed with Fascia Suture Technique (FST).	FST for closure of femoral artery access sites after pEVAR.	Technical success was 91.3% (146/160). 14 failures required cEVAR (mostly due to bleeding). Follow-up showed no signs of increased stenosis or thrombosis. Obesity, femoral calcification, or artery stenosis were not associated with FST failure.
Harrison et al. (2010) [33]	Prospective observational study	38 patients (69 CFAs) undergoing EVAR with devices up to 24 French.	FST for arterial closure following pEVAR.	Immediate technical success was 87% (60/69). The 9 primary failures (due to haemorrhage or thrombosis) were successfully treated with open revision. Pseudoaneurysms occurred in 4 patients but were resolved. Concludes the

				technique is safe and durable.
Melloni et al. (2020) [34]	Retrospective observational study	360 patients (622 femoral accesses) receiving pEVAR for aortic aneurysms.	pEVAR with suture-mediated closure, comparing large-bore (>21F) versus small-bore (≤ 21F) sheaths.	Overall closure success was 94%. After matching, success rates were not significantly different between large-bore (90.7%) and small-bore (93.0%) sheaths. Severe calcification and small arteries (<9 mm) were predictors of failure for large sheaths.
de Souza et al. (2015) [35]	Retrospective observational study	102 patients undergoing complex EVAR (thoracic, thoracoabdominal, pararenal, aortoiliac) requiring large-diameter sheaths.	pEVAR using thoracic, fenestrated, and branched stent grafts, closed with Perclose devices.	Technical success was achieved in 95% of femoral arteries. Access-related complications occurred in only 5% of patients (e.g., thrombosis, haematoma), mirroring rates seen in standard infrarenal pEVAR. There were no 30-day deaths.
Overview of Perclose Proglide [36]	Manufacturer's Website	-	-	Information on Perclose Proglide

Lee et al. (2007) [37]	Retrospective observational study	262 EVAR/Thoracic EVAR (TEVAR) procedures (279 femoral arteries closed with 559 Proglide devices).	Percutaneous access for 12F to 24F sheaths using the "Preclose" technique with Perclose Proglide.	Overall technical success was 94.3% (99.0% for 12F–16F sheaths vs 91.4% for 18F–24F sheaths). pEVAR shortened overall procedure times compared to cEVAR, though savings were offset by the cost of the closure devices.
MANTA Vascular Closure Device [38]	Manufacturer's Website	-	-	Information on MANTA Vascular Closure Device
Prostar XL System [39]	Manufacturer's Website	-	-	Information on Prostar XL System
Lee W.A. (2011) [40]	Textbook chapter	-	Various arterial closure devices compared to manual compression.	Outlines mechanisms, advantages, and failure modes of closure devices. Emphasises that whilst devices offer rapid haemostasis and early ambulation, meticulous technique and patient selection are critical to avoid severe complications like necrotising arteritis.

Perclose Prostyle Overview [41]	Manufacturer’s Website	-	-	Information on Perclose Prostyle
Maniotis et al. (2017) [42]	Systematic review	2,909 patients across published studies undergoing Transcatheter Aortic Valve Replacement and EVAR.	Percutaneous closure using Prostar XL compared to the ProGlide device.	Prostar XL was associated with a higher risk of all bleeding complications (RR 1.82) and major/life-threatening bleeding (RR 2.48) compared to ProGlide. Overall vascular complications, mortality, and acute kidney injury rates were similar.
Uhlmann et al. (2018) [43]	RCT	50 patients undergoing EVAR.	Intra-patient randomisation: pEVAR in one groin and cEVAR in the other.	pEVAR had significantly shorter access times (11.5 vs 24.8 mins). cEVAR resulted in 8 complications in 6 patients, whereas pEVAR had none. pEVAR groins were less painful post-operatively and proved cost-effective despite the added expense of the closure devices.
Read et al. (2024) [44]	Retrospective database analysis	14,002 patients undergoing EVAR for aortic disease (excluding emergencies).	pEVAR compared to bilateral cEVAR, unilateral cEVAR, and failed pEVAR converted to OSR.	Unplanned 30-day readmissions were higher for bilateral (7.6%) and unilateral (7.3%) cEVAR compared to pEVAR (5.7%).

				cEVAR also carried a substantially higher risk of wound complications.
Pratesi et al. (2015) [45]	Prospective observational study	1,322 patients (2,381 access sites) treated by operators with experience of ≥50 procedures.	pEVAR using Prostar XL and Proglide systems.	Overall technical success was 96.8%, with a 3.2% major complication rate requiring conversion to cEVAR. The presence of femoral artery calcifications was the only significant predictor of technical failure.
Melani et al. (2022) [46]	Retrospective observational study	608 consecutive patients undergoing EVAR (373 pEVAR, 202 cEVAR).	pEVAR compared to cEVAR.	pEVAR had a technical success rate of 97.3%. Compared to cEVAR, pEVAR reduced operative time, length of hospital stay, 90-day readmission rates (1.4% vs 6.9%), and early access-related complications (1.6% vs 5.4%).
Bennett et al. (2019) [47]	Retrospective observational study	1,382 patients undergoing EVAR of ruptured AAA.	Locoregional anaesthesia compared to general anaesthesia.	30-day post-operative mortality was significantly lower in the locoregional anaesthesia group (14.6%) compared with the general anaesthesia group (29.2%).

Tay et al. (2022) [48]	Retrospective observational study	35 patients undergoing emergent EVAR of ruptured AAA.	pEVAR, n=21 compared to cEVAR, n=14.	Technical success was 100%. There was no difference in 30-day major adverse events or femoral access-site complications between pEVAR and cEVAR.
Manunga et al. (2013) [49]	Retrospective observational study	222 patients (391 femoral arteries) undergoing pEVAR with 12F to 24F sheaths.	pEVAR using VCDs.	Technical success was 96.4%. Femoral artery calcification (>50% anterior or posterior wall) was identified as a major determinant of failure, whilst body mass index and sheath size were not.
Gradinariu et al. (2022) [50]	Retrospective observational study	511 patients (354 patients in the pEVAR group) undergoing aorto-iliac procedures.	pEVAR compared to cEVAR.	Access-related complications occurred in 11% of percutaneous sites (bleeding was most common at 8.5%). Predictors of complications included low eGFR, increased artery depth, choice of device, and complex procedures. Uncomplicated pEVAR was faster than cEVAR.
Bensley et al. (2012) [51]	Retrospective observational study	299 patients undergoing elective EVAR (168 pEVAR, 131 cEVAR).	Ultrasound-guided pEVAR compared to cEVAR.	pEVAR success rate was 96%, with fewer wound complications (0.7% vs 7.4%) and shorter operative times compared to cEVAR. An access vessel diameter of <5

				mm was a strong predictor of percutaneous failure (OR 7.3).
Ashrafi et al. (2016) [52]	Retrospective observational study	53 patients (96 access sites) undergoing pEVAR.	Introduction of a pEVAR first approach using Perclose Proglide.	Technical success was achieved in 77.4% of patients (86.5% of access sites). Failure was significantly associated with a smaller CFA diameter and >50% circumferential calcification.
Bechara et al. (2013) [53]	Retrospective observational study	99 patients undergoing pEVAR.	pEVAR using suture-mediated VCDs (Proglide or Prostar XL).	Overall technical success was 82%. The probability of closure failure decreased dramatically over time (from 45% initially to 5% at 30 months) due to operator experience. Patient factors (distance from skin to CFA, calcification, sheath size) did not correlate with failure.
Al-Khatib et al. (2012) [54]	Retrospective observational study	120 women undergoing EVAR.	Selective pEVAR using the Preclose technique compared to cEVAR.	51% of the evaluated female arteries met criteria for selective pEVAR, achieving a 96% technical success rate. The cEVAR cohort experienced a significantly higher rate of wound complications (34.8% vs 8.3%), including

				haematomas and pseudoaneurysms.
Liu et al. (2020) [55]	Systematic review and meta-analysis	36 cohorts of patients undergoing EVAR for infrarenal AAA.	EVAR outcomes evaluated by sex (women compared to men).	Women had an increased risk of 30-day mortality (adjusted OR 1.73), in-hospital mortality, limb ischaemia, renal/cardiac complications, and long-term all-cause mortality compared to men.
Marzano et al. (2026) [56]	Systematic review and narrative synthesis	15 studies encompassing >500,000 EVAR procedures.	Standard and complex EVAR outcomes evaluated by sex (women compared to men).	Women consistently exhibited higher early mortality, greater access-related morbidity, and less favourable long-term outcomes, largely driven by anatomical disparities (smaller vessels, hostile vessel necks) and older age.
Thurston et al. (2019) [57]	Retrospective observational study	100 patients undergoing elective EVAR (50 pEVAR, 50 cEVAR).	pEVAR compared to cEVAR.	pEVAR significantly reduced mean operating time (113.9 vs 144.9 mins), overall length of stay (28.3 vs 33.1 hours), and mean hospital costs ($16,628.5 vs $21,705.8).

Mathisen et al. (2026) [58]	Prospective observational study	368 patients eligible for pEVAR.	pEVAR using large-bore access closed with suture-mediated VCDs (ProGlide and ProStyle).	98.6% of groins were successfully closed percutaneously. Primary technical success was 66.4%. Low-profile introducers (12Fr–16Fr) showed slightly better technical success, with no significant difference between ProGlide and ProStyle devices.
Naiem et al. (2022) [59]	Retrospective observational study	170 patients undergoing elective EVAR.	Ambulatory EVAR compared to inpatient EVAR.	Ambulatory EVAR was successful in 84% of planned patients. Total costs at 30 days and 1 year showed a non-statistically significant reduction of 27% and 21%. Cost reductions were statistically significant for males and patients <85 years.
Dosluoglu et al. (2014) [60]	Retrospective observational study	64 patients undergoing elective EVAR.	Ambulatory pEVAR (same-day discharge, SDD) after 6 hours of bed rest.	33% of patients achieved SDD. SDD patients were significantly younger, had smaller AAAs, less blood loss, and shorter operating times. Ambulatory PEVAR was deemed feasible and safe in selected patients.

Kameda et al. (2025) [61]	Retrospective observational study	148 patients undergoing pEVAR.	pEVAR under local anaesthesia compared to prior EVAR methods.	The local anaesthesia group had a significantly higher prevalence of severe renal dysfunction and older age, yet experienced significantly shorter operative times and post-operative hospital stays.
Altoijry et al. (2023) [62]	Retrospective observational study	59 patients undergoing TEVAR/EVAR.	pEVAR (n=35) compared to cEVAR (n=24).	pEVAR resulted in significantly shorter operative durations (141.4 vs 218.5 mins), shorter hospital stays, and lower ICU admission rates. A sheath-to-femoral artery diameter ratio ≥0.74 predicted higher complication and mortality risks.
Krajcer et al. (2019) [63]	Retrospective observational study	1,000 matched patients (250 fast-track EVAR, 750 standard EVAR).	Fast-track EVAR (14 Fr stent, bilateral percutaneous, no general anaesthesia, next-day discharge) compared to standard EVAR.	Fast-track EVAR had shorter operating times, shorter hospital stays, lower intensive care use (4.4% vs 48.0%), fewer major adverse events, and $4,000 lower total perioperative healthcare costs.
Phair et al. (2020) [64]	Retrospective observational study	128 patients undergoing AAA repair (100 EVAR, 28 OSR).	Analgesic prescriptions for pEVAR, cEVAR, and OSR.	EVAR required fewer opioids than open repair. However, opioid prescription rates were similar between pEVAR and cEVAR, though pEVAR

				patients used fewer morphine milligram equivalents during hospitalisation and reported less pain at discharge. Non-opioids were underutilised.
Rebelo et al. (2022) [65]	Retrospective observational study	44 pEVAR and 215 cEVAR accesses for TEVAR.	Minor complications of pEVAR compared to cEVAR.	Overall minor complication rates were similar during hospital stay (11.4% pEVAR vs 9% cEVAR). Bleedings treatable by compression occurred significantly more often in the pEVAR group (6.8% vs 0.5%).
Ķīsis et al. (2012) [66]	Prospective observational study	40 patients with AAA (20 EVAR, 20 OSR).	EVAR using Nellix sac-anchoring endoprosthesis compared to OSR.	Patients undergoing EVAR reported significantly improved health-related quality of life 1 month post-operation (SF-36 score 47 vs 38). Improvement remained slightly better for EVAR at 1 year.
Van Orden et al. (2018) [67]	Retrospective observational study	8,141 patients undergoing exclusively pEVAR.	Local anaesthesia compared to general anaesthesia.	General Anaesthesia was used in 90.7% of cases. After multivariable adjustment, General Anaesthesia was associated with significantly more pulmonary complications (OR 2.8) and prolonged operative time compared to LA. Overall

				mortality and cardiac complications did not differ.
Ioannou et al. (2015) [68]	Retrospective observational study	66 patients undergoing EVAR (median age 72 years, median AAA diameter 58 mm).	EVAR using the Ovation Abdominal Stent Graft System, comparing local anaesthesia to regional/general anaesthesia with a predominantly percutaneous approach.	Technical success was 95%. pEVAR was used in 76% of cases. Local anaesthesia was associated with shorter procedural time (85 vs 107 mins), higher percutaneous access rates (91% vs 45%), and fewer systemic complications (2.3% vs 14%) compared to regional/general anaesthesia.
Harlin et al. (2016) [69]	Clinical study	6 patients (American Society of Anesthesiologists class III) undergoing pEVAR in an outpatient setting.	A novel anaesthetic technique using moderate intravenous sedation (fentanyl) combined with bilateral ilioinguinal nerve blocks.	All patients underwent successful repair without conversion to general anaesthesia. The technique provided excellent postoperative pain relief, reducing the need for narcotics after the night of discharge, and was cost-effective (average anaesthetic supply cost around $7.00).
Lee et al. (2008) [70]	Retrospective observational study	100 patients (accounting for 156 femoral arteries) who underwent pEVAR and had at least a 6-month postoperative CT scan.	Percutaneous closure of femoral arteries using the Preclose technique (with Perclose Proglide devices) following the use of large	The late complication rate related to the closure site was very low at 1.92% (3 out of 156 arteries), consisting of one asymptomatic dissection

			diameter (18F to 24F) introducer sheaths.	and two pseudoaneurysms requiring surgical repair over a mean follow-up of 11.6 months.
Hajibandeh et al. (2016) [71]	Systematic review and meta-analysis	Patients from 3 randomised controlled trials and 18 observational studies undergoing EVAR.	pEVAR vs cEVAR.	pEVAR was associated with a lower frequency of groin infection and lymphocele, as well as shorter procedure times and hospital lengths of stay, without increasing the risk of haematoma, pseudoaneurysm, arterial thrombosis, or dissection.
Liang et al. (2025) [72]	Meta-analysis	28,263 patients diagnosed with type B aortic dissection across 24 studies (14,534 receiving pEVAR and 13,729 receiving cEVAR).	pEVAR vs cEVAR.	pEVAR resulted in reduced hospital stay, decreased operative time, quicker recovery, fewer groin infections, and fewer cardiac complications. However, it exhibited a higher incidence of pseudoaneurysms and surgical suture failures.
Cho et al. (2026) [73]	Retrospective observational study, proof-of-concept validation study	15 patients undergoing elective EVAR matched to 45 conventional control patients via propensity scoring.	Preoperative planning using a physics-informed digital twin and artificial intelligence model to assess the endoleak risk index, compared to conventional static anatomical planning.	The AI model successfully predicted type IA endoleaks with 80% overall accuracy, 100% sensitivity, and 100% negative predictive value, outperforming the conventional planning group (51.1% accuracy). The endoleak risk index was

	higher in patients who developed endoleaks.

AAA, Abdominal Aortic Aneurysm; EVAR, Endovascular Aortic Repair; OSR, Open Surgical Repair; RCT, Randomised Control Trial; pEVAR, Percutaneous EVAR; cEVAR, Femoral Cutdown EVAR; AD, Aortic Dissection; AA, Aortic Aneurysm; CFA, Common Femoral Artery; VCD, Vascular Closure Device; MC, Manual Compression; FST, Facia Suture Technique; TEVAR, Thoracic EVAR;
